# Co-created Mobile Apps for Palliative Care Using Community-Partnered Participatory Research: Development and Usability Study

**DOI:** 10.2196/33849

**Published:** 2022-06-23

**Authors:** Jafar Al-Mondhiry, Sarah D'Ambruoso, Christopher Pietras, Thomas Strouse, Dikla Benzeevi, Armen C Arevian, Kenneth B Wells

**Affiliations:** 1 Division of Medical Oncology Department of Medicine University of Southern California, Keck School of Medicine Los Angeles, CA United States; 2 Division of Hematology & Oncology Department of Medicine University of California, Los Angeles Los Angeles, CA United States; 3 Palliative Care Program Department of Medicine University of California, Los Angeles Los Angeles, CA United States; 4 Department of Psychiatry and Biobehavioral Sciences UCLA Jane and Terry Semel Institute for Neuroscience and Human Behavior University of California, Los Angeles Los Angeles, CA United States; 5 UCLA Clinical and Translational Science Institute Los Angeles, CA United States; 6 Chorus Innovations Los Angeles, CA United States

**Keywords:** mobile phone, mobile apps, mobile health, mHealth, eHealth, digital health, palliative care, quality of life, survivorship, patient advocacy, oncology, patient-reported outcomes, PRO, community-partnered participatory research, CPPR

## Abstract

**Background:**

Open design formats for mobile apps help clinicians and stakeholders bring their needs to direct, co-creative solutions. Palliative care for patients with advanced cancers requires intensive monitoring and support and remains an area in high need for innovation.

**Objective:**

This study aims to use community-partnered participatory research to co-design and pretest a mobile app that focuses on palliative care priorities of clinicians and patients with advanced cancer.

**Methods:**

In-person and teleconference workshops were held with patient and family stakeholders, researchers, and clinicians in palliative care and oncology. Question prompts, written feedback, semistructured interviews, and facilitated group discussions identified the core palliative care needs. Using Chorus, a no-code app-building platform, a mobile app was co-designed with the stakeholders. A pretest with 11 patients was conducted, with semistructured interviews of clinician and patient users for feedback.

**Results:**

Key themes identified from the focus groups included needs for patient advocacy and encouragement, access to vetted information, patient-clinician communication support, and symptom management. The initial prototype, *My Wellness App*, contained a weekly wellness journal to track patient-reported symptoms, goals, and medication use; information on self-management of symptoms; community resources; and patient and caregiver testimonial videos. Initial pretesting identified value in app-based communication for clinicians, patients, and caregivers, with suggestions for improving user interface, feedback and presentation of symptom reports, and gamification and staff coordinators to support patient app engagement.

**Conclusions:**

The development of a mobile app using community-partnered participatory research is a low-technology and feasible intervention for palliative care. Iterative redesign and user interface expertise may improve implementation.

## Introduction

Over the last decade, mobile app technologies for health tracking and support have become widely popular. Most available products come from consumer software companies, with only a minority being generated by health care professionals or research institutions [1]. Few studies have focused on the palliative care needs of patients with advanced cancer, despite high levels of uncontrolled symptoms, including depression, fatigue, pain, anxiety, and distress [2-4]. These needs are compounded by communication issues between patients with cancer and their treating clinicians [4,5]. Given the high cost and health system use inherent to this population [6], innovative technological solutions may help address the care needs that emerge for such patients in ambulatory settings.

Web-based interventions have shown promise in facilitating the self-management of cancer-related symptoms and communication with health care providers [7]. Specifically, the use of web-based platforms to collect patient-reported outcomes (PROs) has been associated with improvements in health-related quality of life and overall survival [8], mediated by proactive management of emergent symptoms. Efforts to integrate artificial intelligence into the design of PRO-collecting apps have resulted in improved cancer-related pain control and fewer pain-related hospital admissions [9].

To date, we are aware of no mobile apps focusing on the needs of patients diagnosed with cancer that have used community-partnered participatory research (CPPR) methods in their development. CPPR is a variant of community-based participatory research that promotes 2-way knowledge exchange and equal transfer of expertise and power sharing with the development of trust among patients, communities, health systems, policy leaders, and academic partners in the planning, process, and products of research [10,11]. CPPR highlights both community and academic shared perspectives, as distinct from community-based participatory research, which primarily focuses on academics supporting the priorities of communities; however, both focus on the importance of collaboration in authentic partnerships [11]. CPPR has been applied across diverse health and social conditions, particularly in underresourced communities, and has been used as the basis for community-level collaborative care interventions in mental health, with evidence of long-term effectiveness relative to standard individual agency training [12-14]. The extensions of this work have supported the collaborative participatory development of mental well-being support apps [15]. Such methods are rooted in the philosophy that the inclusion of patients in the development of interventions aimed at their care may increase perceptions of autonomy and competence in receiving that care, qualities associated with greater medication adherence [16], satisfaction [17], and health-related behavior changes [18,19]. For palliative care populations in particular, such inclusion may enhance a sense of dignity, a core feature of well-being threatened by advanced illness [20], and help normalize palliative care as a core component of health care that merits access to information and support [21]. This quality improvement (QI) initiative explores the experience of using CPPR methods to co-create (phase 1) and pretest (phase 2) a mobile app to meet the palliative care priorities of clinicians and patients with advanced cancer, with a focus on feasibility (inclusion of stakeholders and ease of use) and acceptability (fit with priorities of patients and providers).

## Methods

### Ethics Approval

The University of California, Los Angeles, Institutional Review Board provided expedited review and approval of this QI initiative (phase 1: UCLA#17-000294; phase 2: UCLA IRB#20-002047). Participation in the study was voluntary. Informed consent was obtained from participants at all levels, including focus work group sessions and the pretest study.

### Setting

Study activities were conducted at an academic-community partnership in West Los Angeles, California.

### Phase 1: Participatory App Development

#### CPPR Structure, Planning Committee, and Stakeholder Identification

CPPR uses a *Vision, Valley, Victory* (planning, implementation, and products) process guided by core principles (trust development, 2-way knowledge exchange, respect, partnered development, and equity focus) applied through a structure with a leadership council, stakeholder working groups, and broader input acquired through evaluation. This structure is similar to the *Plan-Do-Study-Act* structure for QI [22], with the distinction that CPPR is driven by iterative stakeholder feedback in every phase of development. In CPPR, the Vision stage can be its own project, including piloting, evaluation, and initial product in preparation for subsequent adaptation and main implementation [10]. Herein, we describe an initial *Vision* phase of development, including the planning, pretesting, and evaluation of an app prototype, created in preparation for a larger implementation initiative. For this project, the health system palliative care QI leadership group invited clinicians from palliative care, oncology, psychiatry, primary care, and urology, in addition to representatives from pharmacy, social work, and health information technology, to join with palliative care patients and family stakeholders to collaborate and explore options to enhance palliative care services with digital technology. Oncology faculty members from the health system outside this leadership committee were recruited to create a core group of 5 physicians and nurse practitioners comprising the provider work group. Using flyers, emails, and direct patient outreach, 4 patients receiving oncology and palliative care services in the same health system were recruited for the patient work group. A fifth patient representative was recruited as a diversity leader with a family history of advanced cancer and palliative care exposure within the same health system.

#### Participatory Technology Development Platform

The goal of this planning QI (*Vision*) initiative was to collaborate with provider and patient-caregiver working groups to develop a mobile app to address the diverse needs of clinician and patient stakeholders in the delivery of high-quality palliative care for patients with advanced cancer. Chorus is a no-code app-building platform that enables individuals without technical training to use a simple, visual web interface to create interactive web-based apps optimized for mobile app use, accessible by computers or smartphones [15]. Consistent with the principles of CPPR, this allows both patients and clinicians to be involved in all aspects of product development, with previous success documented using this platform in ethnically diverse urban populations [15]. This technology allows users to create, test, and modify mobile app content in real time, with no programming experience required.

#### Work Group Structure and Feedback

**Figure 1:**
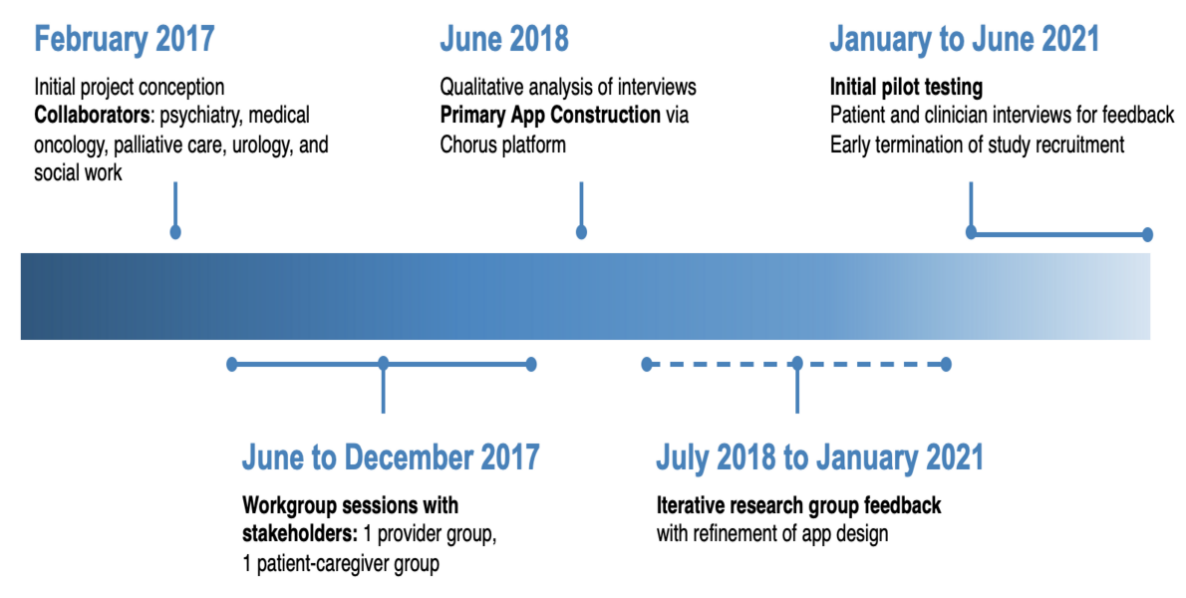
Timeline for app development.

**Figure 2:**
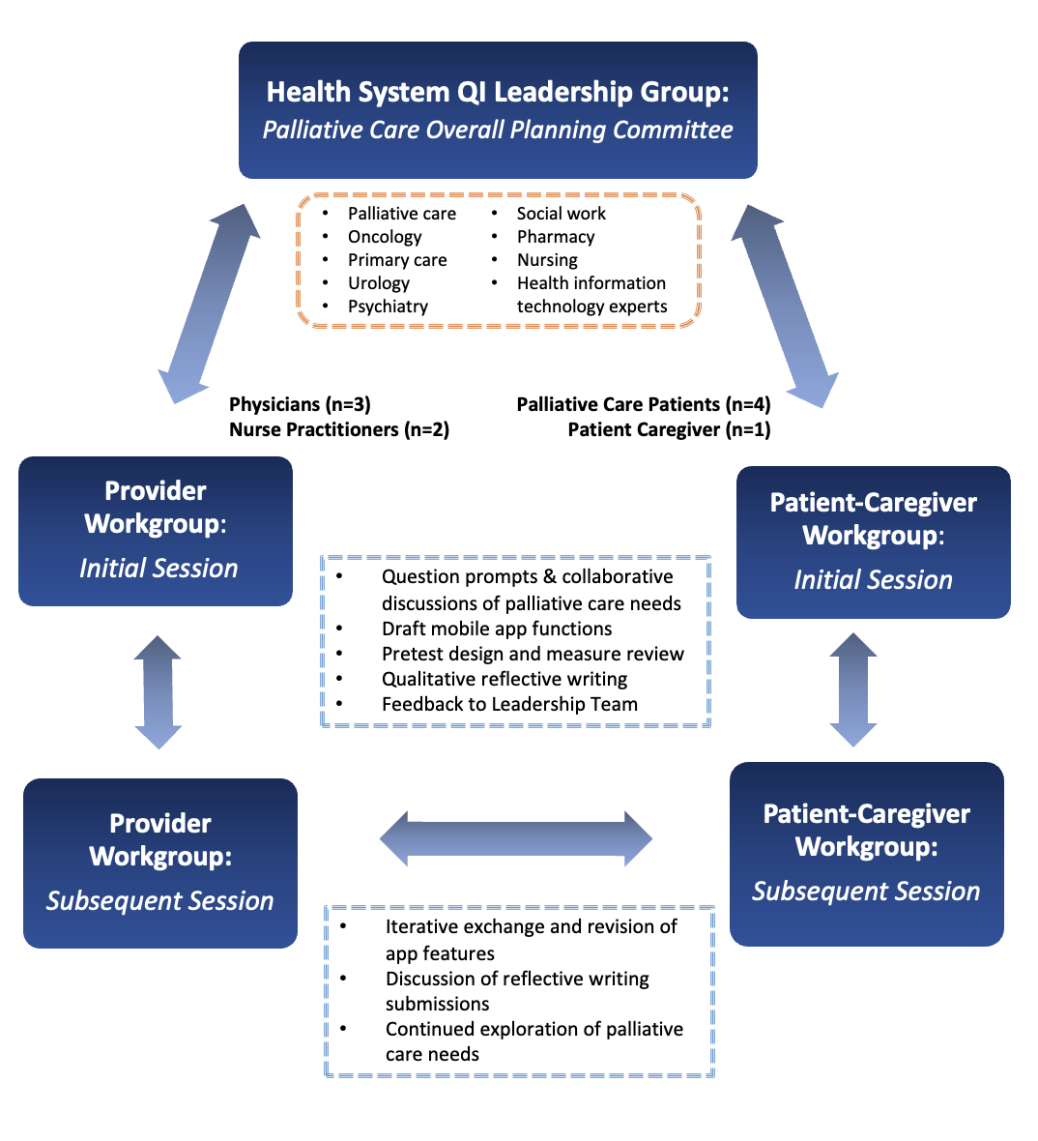
Work group structure and feedback cycles. QI: quality improvement.

From June to December 2017 (Figure 1), weekly provider and patient work group meetings were conducted for a total of 4 sessions per work group. Work groups were facilitated by 2 members of the QI leadership team (AA and KB). Following CPPR principles [10], the work group participants were given an orientation in the CPPR methods and Chorus app features and asked to share their experiences and perspectives. Question prompts were offered to elicit discussion. Participatory development with Chorus involved an iterative cycle of four steps: (1) identifying key barriers and opportunities for improving palliative care delivery using a mobile app tool, (2) generation of app content, (3) creating working prototypes of the mobile tool in real time by QI leads using Chorus, and (4) testing draft mobile apps in real time during workshops with iterative improvement of tools based on feedback from the work group (Figure 2). Modifications in app development continued until a consensus was reached among the stakeholders. Consistent with the principle of 2-way communication and knowledge exchange, a patient stakeholder participated in the provider work group and vice versa. Standardized reflective discussion prompts were administered at the end of each work group session to prompt discussion of process and progress and to inform agendas for subsequent sessions (Multimedia Appendix 1). Minutes were taken by the session leaders and support staff. For each work group (provider and patient-caregiver), one main overview session for each work group before the main app development was audio recorded and transcribed for subsequent qualitative analysis to illustrate the work group process. To facilitate feedback effectively and efficiently, we used rapid analysis techniques [23] to synthesize themes from work groups into generalized categories supported by representative quotes from transcripts or meeting notes. Notes, audio recordings, and transcripts from each work group were reviewed by 2 members (JA and KW) to reach an agreement on concepts and themes and select representative examples. Patient and family member participants were offered US $20 gift cards for taking part in each of the 2-hour work group sessions, in addition to parking validation.

### Phase 2: Pretest

To determine the feasibility and acceptability of the initial product, the *My Wellness App*, we conducted a pretest, with the goal of recruiting at least 10 patients via a blanket email invitation to the entire palliative care patient panel of one of the authors (SD). All recruited patients were undergoing care for metastatic cancer, were aged at least 18 years, had English-language proficiency, and had access to email and a mobile smartphone. To elicit views independent of the developers, pretest participants were not involved in designing the app. Baseline surveys were administered to measure digital health preferences (developed by the work groups), a Brief Pain Inventory-Interference scale [24], and abbreviated Patient Empowerment Scales [25] (Multimedia Appendix 2). Consistent with CPPR, all scales were chosen with input from patient and provider stakeholders, with the goal of having standardized measures relevant to both parties. A provider portal was developed to allow mutual access to content shared on the app by patients with their treating clinician. The consenting clinician from the planning group provided app orientation to the participating patients, encouraged weekly *Wellness Journal* entries, and reviewed patient input via the shared provider portal during regularly scheduled clinic visits or by telephone, as indicated. Semistructured interviews were conducted and recorded with the treating clinician (SD) and a patient to elicit feedback for improvement.

## Results

### Phase 1: Participatory App Development

#### Patient Work Groups

Drawing from their own experiences, the patients identified several goals for an app to support palliative care delivery (Table 1). One key theme mentioned during work group discussions was the difficulty in finding well-vetted material regarding their medical problems and symptoms; for example, “I think the information is out there, but it is in a sea of misinformation.” Another key theme was the need for a balance between having more information on medications, supplements, herbs, drug interactions, and side effects with the need for streamlined and easy-to-read information. The third theme was the elusive nature of symptom management, with priorities shifting from day to day and difficulty knowing how to communicate with their care team appropriately. Patients sympathized with the needs of their clinicians, wanting to provide accurate and well-synthesized information; for example: “Every individual has their own reference of how serious the side effect is, and not everyone expresses it in the technical language that would be most useful for a provider.” In addition to offering information to their providers, a fourth theme was that patients acknowledged that tracking symptom scores could have a personal benefit in helping to reflect on changes, better understand symptoms, and potentially know when to act. The fifth theme was placing symptoms in a functional context (ie, what they mean and how to moderate them) to help patients both demystify and get perspective on the impact of their symptoms and through that build confidence and self-efficacy in symptom management.

**Table 1:** Themes from patient work group sessions on app development

Theme	Example
Peer-to-peer descriptions of palliative care (dispelling misconceptions)	“The oncologists don’t necessarily communicate [about palliative care] and then people get really scared of it. I’ve seen it, a lot of friends of mine who are newly diagnosed and I’m trying to guide them as someone who’s been sick for so long.” “My reaction was I don’t want to do palliative care ... my initial reaction was this is end of life. And I didn’t understand what it was at all.”
Tips in understanding and managing symptoms	“If I had put [my symptoms] into the system, and then I could see a timeline, a graph of it, I may have gone in sooner knowing, well I’m fooling myself. I’ve had, you know, 15 days of severe pain. I need to do something about it.” “It’s so important, I mean, managing side effects enables you to get more treatment.”
Tracking and encouraging progress	“‘Thrivership’ in my community, which is the metastatic breast cancer community, it’s very crucial word because what we read out there is very depressing and a downer.”
Improved patient-clinician communication	“If we’re going to use pain scales, put it in context. Because I know sometimes when I’m asked, ‘What’s your pain?,’ I feel a lot of pain. But if I really think about it, you know, I drove today, so I’m in pain, but I’m able to drive or I walk the dog. I’m like, yeah, I didn’t feel good but I was able to walk the dog, so it’s not that horrific.” “Every individual has their own reference of how serious the side effect is, and not everyone expresses it in the technical language that would be most useful for a provider.”
Building confidence	“It’s a bit of perspective. So having those kinds of trigger questions [around symptom context and daily activities] may make patients to refocus on what’s good.”
Vetted information about medications and herbs	“I think the information is out there, but it is in a sea of misinformation.” “People are out there and they’re Googling and looking for that, and they’re getting scared. And many of these [health] systems don’t have an official list of our recommended resources.”
End-of-life care planning	“I think that a way to help patients get through that [end-of-life planning] process may be information. It’s very complicated.” “I think that’s such an important issue that people are so scared of, because it causes all sorts of family fighting ... maybe just having an advance directive tool up on the site, having that paperwork available.”
Improved patient advocacy	“Explaining why [you should] have someone with you, why record your [clinic] sessions, why bring a list of questions, you could say that studies show that the recall rate after leaving a doctor’s office is at best 30%.”

In addition, although patients in this work group were engaged in palliative care, they were all aware of the stigma surrounding palliative care as a term often misunderstood to describe only end-of-life care or hospice care, rather than surviving and thriving despite serious illness. As a result of this input, suggestions were made for the app to address a more expansive definition of palliative care as a positive service that provides supportive care and symptom or side effect management from diagnosis onward, highlighting the benefits of palliative care in improving patient quality of life while in active treatment. To proactively address stigma and normalize the delivery of palliative care, QI leaders and patients collaboratively suggested including peer-to-peer engagement in the form of video testimonials on the app. Empathizing with the needs of other patients recently diagnosed with cancer, patient stakeholders wanted an app that addressed challenges in survivorship, such as how to have successful encounters with their clinicians, with advice delivered to app users in laymen’s terms. Videos from patient peers explaining how to advocate for oneself, how to recruit help from caregivers during appointments, and what to expect from palliative care were suggested as solutions. In addition, patients requested vetted lists of resources to help them quickly navigate their changing needs and help with end-of-life and advance care planning. Ultimately, they felt the app features needed to be rich and adaptable to each patient’s needs, with opportunities for growth and customization: “Something that I learned being sick over these last three years is that every single person, even with the same diagnosis handles it differently.” Stakeholders requested that the videos would represent diverse populations and experiences with palliative care, including clinicians, patients, and family members.

#### Provider Work Groups

Several consistent themes and goals were developed over the course of the provider work group sessions (Table 2). Concerns around app time demands from patients and providers were balanced with the hope of creating greater efficiency in patient-provider communication. Providers acknowledged the difficulty patients had in discussing symptoms and worked to create app functions that measure symptom severity, track progress, and alert providers when threshold symptom levels are met. The goal of improving communication was the dominant theme, using the app to streamline patient symptom trends between visits and triage emergencies. A specific focus was placed on pain as a key symptom affecting the quality of life and the creation of a comprehensive pain diary to allow patient reflection on aggravating or alleviating factors and context. Providers were interested in how the app could elicit a patient’s medication use patterns, promote adherence, and provide a way to review and address symptoms. Providers were sympathetic to the emotional and socioeconomic impact of cancer and hoped to provide patients with tools to address these barriers to care through encouragement and a curated list of local resources. The app was suggested as a potential anchoring point for caregivers, allowing them access to symptom reporting on behalf of the patient and symptom management tips, features that may ease their anxiety while advancing the patient’s care. This was also seen as a support for patients with advanced symptoms who may not have the capacity to manage their own needs or communicate effectively with their clinician.

**Table 2:** Themes from provider work group sessions on app development

Theme	Example
Comprehensive pain diary	“If you’re going to do the pain score, tracking, well, when the pain was this—what did I do for it? Or what did I take?”
Medication reminders and use monitoring	“If you’re working with a patient, you can say, well, how often do you feel you need a reminder? And then do you have the flexibility of every day, every other day?”
Provider alert messaging	“If it [patient pain level] meets a certain threshold, then you know, then there’s a prompt to send out an alert.”
Improved triage and communication	“The two things I hear from people all the time is: it’s hard for them to describe things, and they don’t know what to do when to call, when to panic. And if you address those two—you say, okay, if your pain gets to a five, I need you to pick up the phone—then they relax.” “Because then when they call, you’re like, oh, let me have a look [at patient generated symptom reports on the app] and see what you’ve done. Ok, that didn’t work. Ok, that worked. Oh, I can try this.” “If we’re going to change somebody’s pain medication, it would be great to know what the last four or five days looked like.”
Addressing barriers to care	“Resources that are kind of all scattered across the internet, consolidating those resources in one place and have those be resources that are vetted.” “We can broaden the application so that not only do we manage symptoms that are disease related, but also any impediments to the delivery of care. That’s really important because I mean, to get people to comply with what you propose...” “We probably should include some kind of either logistical or financial scale, because I think sometimes those issues contribute to compliance and symptom management, but patients are not prompted and won’t volunteer that.”
Encouraging and supporting patients through difficulty	“I think when I first get someone who’s newly metastatic, it’s a whole shift in psyche ... And when they progress on their therapy, even though they’ve been through it once they’re like, oh crap, I’m back at the beginning again. And so how do we get them moving through that next set of events and getting that [next] therapy started. Because they’re sort of on this constant roller coaster, emotional and physical and psychological. Living scan to scan.”
Vetted educational material for patients	“We’re always telling patients, ‘don’t look at that—look at this.’”
Partnering with caregivers	“By the time they’re at that point [of serious symptoms], a lot of times the caregiver is involved. And I think the caregivers really calm down if you give them stuff to work from, like a document. I think that’s really important because most of them feel so inadequate.”

#### App Design

**Figure 3:**
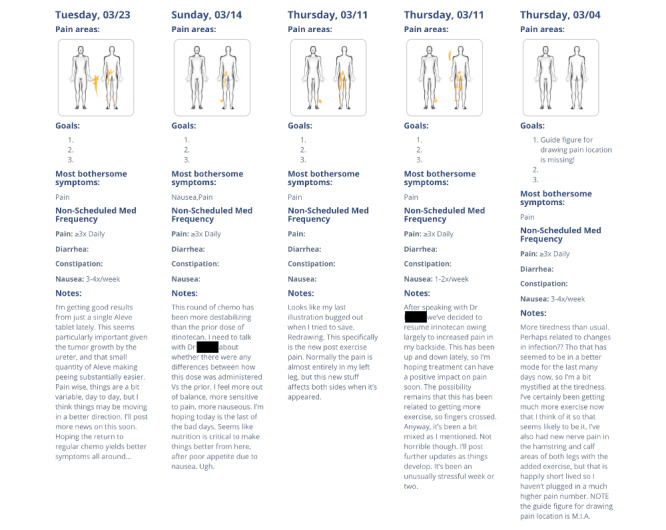
Example of My Wellness Journal details.

**Figure 4:**
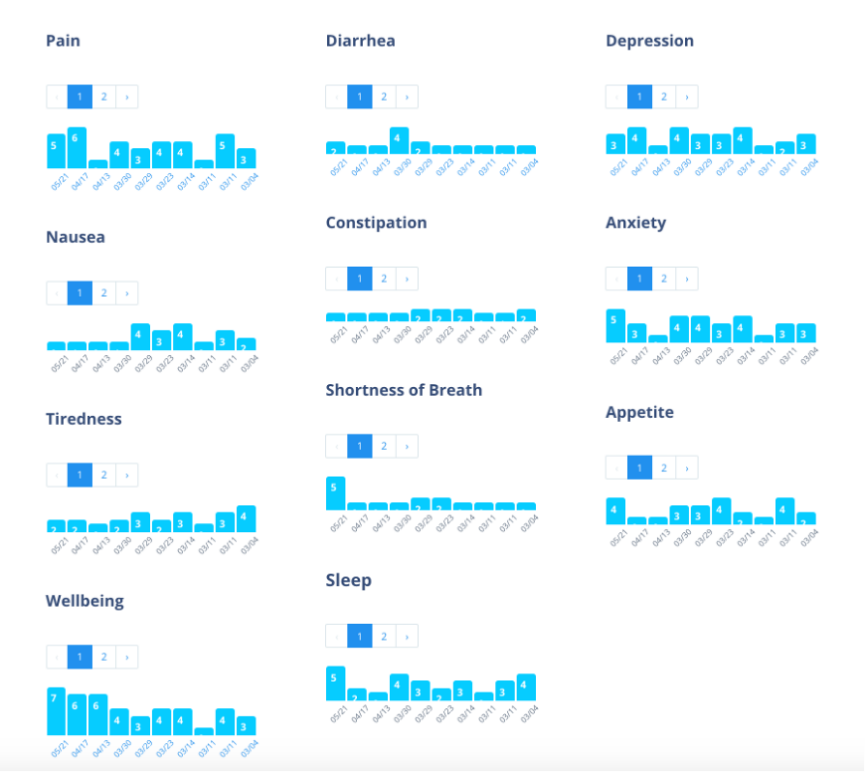
Example of modified Edmonton Symptom Assessment Scale representations.

On the basis of iterative stakeholder input and review, the initial *My Wellness App* prototype contained four core features: (1) a *Wellness Journal*, where patients are encouraged to make weekly entries; (2) *Tips* & *Tools* for symptom and medication self-management; (3) *Voices of Palliative Care*, where brief videos of palliative care clinicians, patients, and caregivers can be viewed; and (4) a *Resources List*, where a directory of local patient support resources can be viewed, including links and information to access transportation, home services, hospice, and legal and insurance benefits. To enhance patient and clinician communication, the *Wellness Journal* contained a modified Edmonton Symptom Assessment Scale (ESAS) [26], a body map for visualization of pain, areas to report medication use frequency, goal setting, and open diary free text entries to permit direct patient *voice* (Figure 3). ESAS entries were displayed longitudinally to track trends and were available for both patients and their treating clinicians (Figure 4). The initial app design encouraged weekly entries with automated email reminders sent to nonresponders each week. Owing to technical limitations, no integration with the existing electronic medical records or alert messaging for symptom score thresholds was included in the initial pretest phase. The *Tips & Tools* feature included brief information reviews for common issues with links to videos and more in-depth reviews and resources. Specific topics included symptom management (eg, nausea, diarrhea, and constipation), coping with difficult emotions, mindfulness, nutrition, and the use of herbs and botanicals, all areas suggested by stakeholders. The *Voices* section included members of the provider and patient work group, offering videos of patient introductions for *What is palliative care?* and other features of the app, the importance of symptom monitoring, stories of survivorship, and tips for maximizing support and clinician communication. One area of shared concern for patients and providers was addressing the stigma of palliative care through effective communication. Feedback from a patient is as follows: “Do you want to keep using the term palliative care, cause it scares people. I mean, like I said, it scared me.” An example from a patient in the provider group is as follows: “The advanced stage breast cancer survivor has a negative connotation. If you want to expand this to a greater cancer or illness community, then maybe there are other words that connect with a greater population.” A provider’s response was as follows: “So I think we have to rebrand this, so people see it as a vital tool that ensures their success during their treatment.” This shared concern led to the proposed app name, *My Wellness App*.

#### CPPR Process for App Development and Pretest Design

Throughout the stakeholder work group sessions, multiple demonstrations of CPPR principles occurred [10], summarized by key themes and examples of quotes from transcripts of key work group sessions at the transition from initial planning to app drafting (Tables 3 and 4). Consistent with the principle of coequal leadership and power through 2-way knowledge exchange, the design of the final app product was equally informed by patient and provider stakeholder input. The protection and elevation of vulnerable participant voices is a core feature of CPPR; to avoid the threat of power imbalances in participation, separate work groups for providers and patients provided secure platforms for interaction in the design process. The 2-way knowledge exchange was illustrated by the rich interaction between academic leaders and individual work groups (see examples in Table 3). Additional knowledge exchange was made available by the inclusion of patients within the provider work group and vice versa. The principle of trust development was evident in the openness of patients to share vulnerable experiences about symptoms, concerns with the dying process, and conflicts experienced with health systems and providers. Providers also demonstrated trust in disclosing their frustrations with patient care and health systems. Respect was evidenced by repeated invitations for stakeholder perspectives as well as agreement and expansion by patients of academic leader suggestions (eg, creating videos). Partnered development took place through the interaction of academic leaders and patients in defining goals (such as having informational resources and access to a shared portal) and reviewing images and options for videos (recording of patient stakeholders). The principle of equity was pursued through the inclusion of a patient caregiver representing ethnic diversity, and attention was paid to assessing financial barriers. In addition, stakeholders commented on the potential needs of vulnerable populations, for example, providing navigator or caregiver support tools for older adults with technology access limitations.

**Table 3:** Community-partnered participatory research process in patient-caregiver work group at transition to app development.

Principle		Example (quote)
**Coequal leadership and power**		
	Attention to patient-provider information and power imbalances (eg, creation of platforms for app design and engagement) Input of patient and family stakeholders on app design features	“I think it would be helpful to have a way to get information about your diagnosis and the chemo.” “[Establishing] a way to email a doctor where you don’t have to know his email address.”
**Two-way knowledge exchange**		
	Rich interaction of academic leaders and patients Inclusion of patients in provider work groups (and vice versa)	Patient: “The advanced stage breast cancer survivor has a negative connotation.” Provider Response: “So I think we have to rebrand this, so people see it as a vital tool that ensures their success during their treatment.”
**Trust development**		
	Frequent exchange of vulnerable experiences Disclosure of conflicts with health system and providers by patient-caregiver stakeholders	“It’s not that you’re dying. You’re about to die. So you need to have an advanced directive. People need to know what your wishes are.” “I had a really painful morning. And then I did my activities during the day and I forgot about the morning, That’s something that the doctor needs to know. But they’re not clear on that.”
**Respect**		
	Repeated invitations for stakeholder perspectives Agreement and expansion by stakeholders of academic leader suggestions	Leader: “[We’re] hoping we could think through how we might be able to help the experience of individuals in palliative care.” Leader suggestion: track “how’s your pain going?” Patient response: “That would be actually very helpful because you don’t know what you don’t. I think that’s actually a really good idea.”
**Partnered development**		
	Interaction of academic leaders and patients in defining goals (eg, creating informational resources and access to a shared portal)	Technology lead: “Do you think you would want a summary screen?” Patient response: “I think for me, a little journal, I keep it on my phone. If this was on the system, then I wouldn’t have to think, how many days was there pain, you know, moderate pain or severe pain.”
**Equity**		
	Inclusion of multiple patients and caregiver with racial and ethnic diversity Attention to potential disparities	“I learned being sick over these last years that every single person, even with the same diagnosis handles it differently.”

**Table 4:** Community-partnered participatory research process in provider work group at transition to app development.

Principle		Example (quote)
**Coequal leadership and power**		
	Input of provider stakeholders on app design features Attention to patient-provider power imbalances (eg, creation of platforms for app design and engagement)	Provider: “If a patient has pain and they’re on pain medication, they could potentially be inputting their pain symptoms on a daily basis into this web app. And that gets fed back to the clinician at a regular visit.”
**Two-way knowledge exchange**		
	Rich interaction of academic leaders and providers Inclusion of patients in provider work groups (and vice versa)	Provider: “The other thing I would love is a, a calendar that records things for the patients, because we constantly use that as a way to track therapies as well.” Technology response: “Manual would be a way to do that, to see if it’s helpful and if it works and we can sort out to automate.”
**Trust development**		
	Frequent exchange of vulnerable experiences Disclosure of conflicts with health system and patients by each provider stakeholders	“I don’t know if I can do that for every patient. Do we expect them to actually be logging into the website?” “Physician or practitioner who is having to go into their inbox multiple times a day, dealing with everything that comes through and there’s no priority currently.”
**Respect**		
	Repeated invitations for stakeholder perspectives Agreement and expansion by stakeholders of academic leader suggestions	Technology lead: “Is it possible to help improve aspects around palliative care with technologies like apps, could they be tailored and created in a way that might address problems that we are having either as providers or from the patient’s perspective or caregivers of palliative care?” Provider: “I mean, I welcome more data rather than less. As long as they can do it.”
**Partnered development**		
	Interaction of academic leaders and providers and patients in defining goals (eg, creating informational resources and access to a shared portal)	Provider: “I think when I first get someone who’s newly metastatic, cause it’s a whole shift in psyche. And how you treat them, getting started on new therapy. And even when they progress, even though they’ve been through it, I’m back at the beginning again. And so how do we get them moving through that next set of events and getting therapy.” Technology response: “If you’re working with a patient, you can say, well, how often do you feel you need a reminder? And then do you have the flexibility of every day, every other day? And if they are going to forget, you need a family reminder.”
**Equity**		
	Inclusion of patient caregivers and members representing ethnic diversity Attention to potential disparities	“We probably should include some kind of either logistical or financial scale, because sometimes those issues contribute to compliance and simply naturally, but patients are not prompted well, to be able to afford their treatment.” “One of the things we have to be careful because if we don’t we create the system that others cannot access and we are inadvertently discriminatory.”

Similarly, CPPR principles were followed in developing the pretest study by reviewing plans and gathering input studies from the patient-caregiver and provider work groups. An example of enthusiasm from a patient stakeholder is as follows: “I think you’ll find a lot in the beta testing as you get information back.” Providers considered who should be included in the pilot, after a QI lead asked the following question: “Is it people who are relatively early with minimal symptoms, or later on, more advanced?” The provider stakeholder response was as follows: “I think you could get both. I mean, I have some patients that come in newly metastatic or in acute symptom crisis until we can get their disease under control that, and then we have some that are asymptomatic with their disease.” Stakeholders also gave input on the selection of items and measures.

### Phase 2: Pretest Findings

A total of 11 patients completed the consent forms and were registered with the app, representing a diversity of cancer diagnoses (n=4, 36% breast cancer; n=3, 27% lung; n=2, 18% colorectal; and n=2, 18% other) and ages (median 58, range 49-82 years; Multimedia Appendix 3). Of these patients, 9 (82%) completed the baseline surveys. The patients identified predominantly as “Caucasian” (8/9, 89%), female (6/9, 63%), held either a 4-year (5/9, 56%) or postgraduate degree (3/9, 33%), and had high composite pain scores on the Brief Pain Inventory-Interference scale (average 6.54). Over the first 6 months of the pretest study activity, 3 patients regularly participated in weekly journal entries, with the other 6 completing one or none during the entire study period. The average composite pain scores between high app users and nonusers were comparable (6.42 vs 6.59). Feedback in the form of semistructured interviews with the treating clinician for all study patients, as well as with one regular user of the app, revealed several key insights (Textbox 1). Overall, it was noted that the completion of surveys, journal entries, and interviews was limited by the high symptom burden of patients, with 2 participating patients dying shortly after the study period.

**Themes from clinician and patient feedback during pretest study.**

**Clinician feedback**

- Value in body mapping
- Lack of app navigator support
- Response variability (differences in numeric scale reporting)
- Opportunities for caregivers
- Higher symptom burden: higher benefit from app use

**Patient feedback**

- Sense of preparedness in symptom communication
- Limited patient onboarding (confusion about app functions)
- Lack of feedback and reinforcement
- Desire for improved user interface
- Log-in friction (absence of home screen app icon)

From the patient’s perspective, a lack of formal onboarding to the app left them unaware of many core features, including goal setting and tips for self-management. Patients expressed a desire for a better app interface, specifically for data representation and interpretation of those data in terms of important trends or changes, as well as symptom management suggestions. A user derived motivation to use the app because of the connection it brought them with their clinician and that participation via the app made them a *good citizen* of the clinic: “I like the promise of it ... and knowing the right information to talk about when we meet.” For most, however, motivation was a challenge, citing a lack of feedback and encouragement from the app itself to continue inputting data. Suggested solutions included gamification of inputs, with different milestones for completion, coupled with haptics upon each submission. As a web-based app formatted for mobile phone use, the app itself was not one that users could find in an app store or have loaded as an icon on their home screen, a detail that bothered some users and created an additional point of friction in accessing the app more quickly or regularly. Using this more familiar format was suggested as having potential for creating push notifications and visual cues over the icon to remind users of outstanding tasks to complete as part of being a patient user.

From the treating clinician’s perspective, the lack of a patient navigator created several challenges. First, it increased the amount of time required by the provider to orient each patient to the app and its features. Second, it placed the onus on the clinician to expeditiously review and respond to all *Wellness Journal* entries that might signal uncontrolled symptoms. Creating threshold symptom level scores for automated messaging to the clinician was proposed as a potential solution; however, at a minimum, this would create an added layer of work for the clinician, and significant variability in symptom reporting for each patient made this potentially challenging (eg, some patients regularly report pain at an 8 out of 10, requiring frequent responses). Similarly, providing personalized reminders or check-ins that could increase motivation and adherence was left to the treating clinician.

App features that were of particular value included the body map, which helped quickly identify changes in the nature of pain symptoms and provided a meaningful jump-off for clinical exploration. Trends in app use and value to the clinician seemed to correlate with patients who had a higher symptom burden from disease activity, changes in treatment, or both. Finally, as predicted in the work groups, caregivers of patients enrolled in the study found value in filling out *Wellness Journal* entries with the patient, both as a way to formally check in with one another and to become more actively connected to the patient’s care by communicating with their clinician.

## Discussion

### Principal Findings

Herein, we describe the first known report of applying CPPR structure and principles to co-create (phase 1) and pretest (phase 2) a mobile app based on patient and clinician stakeholder needs for palliative care delivery, as part of the *Plan* phase of QI initiative and *Vision* phase of CPPR [10,22]. Several examples of web-based and mobile technology-based interventions to elicit PROs and improve patient-provider communication exist in the literature [27-35]. However, in this study, we describe a process of identifying and addressing needs at a local level through a QI initiative, with stakeholders co-leading the app design and pretest process, following CPPR principles of trust, respect, 2-way knowledge exchange, and coleadership [10]. Implementation in our study also follows from the framework of the self-determination theory, which states that behavior is driven by 3 primary psychological needs: autonomy, competence, and relatedness [36]. By structuring interventions around the needs and input of local stakeholders (relatedness), promoting patient engagement in the process of weekly symptom journaling and goal setting (competence), and eliciting feedback for immediate app improvements and tailoring to local group needs (autonomy), we believe our study followed this model in conjunction with CPPR. However, the ultimate goal of patient engagement is to activate health-related changes and participation in the process of medical care, goals reserved in this case for a follow-up implementation and evaluation process (*Valley*) informed by suggestions for app design improvements from the initial planning (*Vision*) phase. Pretesting suggested the feasibility of some engagement in app use while revealing the need to enhance design and engagement, particularly given the high clinical needs in the patient population as well as the time limits of providers.

Accordingly, the next steps for development may include the incorporation of a patient navigator to assist in the management of patient-generated data and improve the process of providing timely and personalized follow-up for changing symptomatology, while reducing provider time burden in explaining and monitoring app engagement. Data summaries and suggestions for clinicians and patients in the management of symptoms could be enhanced using artificial intelligence to generate clinician support recommendations, as demonstrated in previous studies [9,30]. This may serve the expressed desire from patients for increased communication from the app, providing cues for continued participation and streamline information review for clinicians. Expansion of recruitment to include multiple clinicians and a larger cohort of patients at the next stage could provide additional iterative feedback for app redesigns as part of the 2-way knowledge exchange [10].

We believe our QI planning (*Vision*) initiative and pretest suggest that the co-creation of a mobile app care is a feasible, low-technology strategy for potentially improving the delivery of palliative care while lowering the bar for patient and clinician participation in such initiatives. However, our pretest suggests that such app products require continued improvements, creativity, and skills in app interface design to enhance patient motivation and support participation. Although not specifically measured in this study, we may borrow the language of the Technology Acceptance Model framework [37] in describing that clinician and patient app users in phase 2 identified significant perceived usefulness for this app but also pointed out areas where perceived ease of use could be improved. Perhaps out of necessity, most patients with advanced cancer receiving palliative care have already established other mechanisms for symptom reporting and clinic contact (eg, telephone, email, and patient portal messaging). New technologies, such as apps, need to offer even greater convenience and user gratification than these currently available channels, which may then engage patients to explore other content included from stakeholder input. Gamification, haptics, and smartphone icon interfaces are examples of features that may not be immediately recognized or implemented by stakeholders unfamiliar with app design; however, such features may significantly complement and motivate the app experience. Implementation at this level will require additional partnership with technology leaders familiar with these forms of app development, coupled with orientation and training of patient and provider stakeholders to co-create these features, an approach similar to that used in developing a COVID-19 wellness website integrating stakeholders and technology input [38].

### Limitations

The limitations of this study include the preliminary pretest phase of development, with a small number of patients enrolled from a single institution and a single coordinating provider-investigator. As a QI effort using stakeholder-partnered development and evaluation, the results are, by design, meant to be reflective of the local community and thus may not reflect the needs of other communities, regions, or health systems. Furthermore, we acknowledge that the qualitative nature of the data generated from planning and small stakeholder groups may limit generalizability, even within this community. The authors hope that future expansion into other settings and populations, as well as attending to this initial feedback, will enrich the understanding of palliative care needs for patients with advanced cancer through iterative and diverse stakeholder inputs. Additional limitations in the pretest include our focus on English-speaking patients and those with smartphone access, which potentially excludes some underresourced populations.

### Conclusions

Overall, this planning initiative and pretest reinforce the feasibility of applying the CPPR framework to stakeholder co-created palliative care apps, with recommendations identified to more consistently and effectively support patient-caregiver use of the app with their clinicians.

## Appendix

Multimedia Appendix 1 Work group reflection sheet.

Multimedia Appendix 2 Baseline survey for pretest study participants.

Multimedia Appendix 3 Pretest study patient characteristics.

## Abbreviations

CPPR: community-partnered participatory research
ESAS: Edmonton Symptom Assessment Scale
PRO: patient-reported outcome
QI: quality improvement
