# Supplementary material for: Co-created Mobile Apps for Palliative Care Using Community-Partnered Participatory Research: Development and Usability Study
Source: JMIR Form Res. 2022 Jun 23;6(6):e33849. doi: 10.2196/33849 (PMC9264134; doi:10.2196/33849)
Supplement: Multimedia Appendix 1 [file formative_v6i6e33849_app1.docx]

TELL US WHAT YOU THINK?

Date_______________

1. Please tell us what DID work and what DID NOT work in today’s meeting (e.g., quality of discussions, working toward agreements, trust/vision building, planning activities)?
   1. What DID work:
   2. What did NOT work:
2. What new events, activities, strategies, tools, partnerships, technical assistance were a part of the discussion?
3. Please tell us any lingering issues/questions you want addressed at the next meeting.
4. Is there additional information/support you need?
