# Supplementary material for: Co-created Mobile Apps for Palliative Care Using Community-Partnered Participatory Research: Development and Usability Study
Source: JMIR Form Res. 2022 Jun 23;6(6):e33849. doi: 10.2196/33849 (PMC9264134; doi:10.2196/33849)
Supplement: Multimedia Appendix 2 [file formative_v6i6e33849_app2.docx]

**Survey Questions**

**Basic Demographics (3 Questions)**

1. What is your marital status?

- Married
- Widowed
- Divorced or separated
- Single, never married
- Living with someone (as though Married)

1. What is your racial background?

- American Indian/Alaska Native
- Asian, includes East Indian
- Native Hawaiian or other Pacific Islander
- Black or African American
- White/Caucasian, not of Hispanic/Latino descent
- Hispanic/Latino
- Something else
- Don’t know

1. What is the highest level of school you have completed or the highest degree you have received?

- Less than high school graduate (including Grade 12 with no diploma)
- High school graduate (Grade 12 with diploma or GED certificate)
- Some college, no degree (includes some community college, associate's degree, etc.)
- Four year college or university degree/Bachelor's degree (e.g., BS, BA, AB)
- Postgraduate or professional degree, including master's, doctorate, medical or law degree (e.g., MA, MS, PhD, MD, JD, graduate school)

**Digital Health Values/Practices (13 questions)**

1. Overall, how interested would you be to learn more about how to use mobile technologies to improve your health?

- Not at all interested
- A little bit interested
- Moderately interested
- Very interested

*Which of the following statements most matches how you feel about your cell phone, even if neither one is exactly right?*

- Not always need
- Couldn't live without
- Freedom
- Leash
- Connecting
- Distracting
- Helpful
- Annoying

*How often do you use a computer for the following tasks? (Please check one response per Item)*

1. To receive or send email

- Never
- Less than Once per Month
- Monthly
- Weekly
- Daily

1. To order goods or services

- Never
- Less than Once per Month
- Monthly
- Weekly
- Daily

1. To read about general health information

- Never
- Less than Once per Month
- Monthly
- Weekly
- Daily

1. To read information about medications

- Never
- Less than Once per Month
- Monthly
- Weekly
- Daily

1. To read about my health conditions (for example, blood pressure, diabetes, or weight)

- Never
- Less than Once per Month
- Monthly
- Weekly
- Daily

1. To track my health conditions

- Never
- Less than Once per Month
- Monthly
- Weekly
- Daily

1. To track my diet or exercise

- Never
- Less than Once per Month
- Monthly
- Weekly
- Daily

1. To find out about services offered for my health conditions

- Never
- Less than Once per Month
- Monthly
- Weekly
- Daily

**BPI/Brief Pain Inventory (7 questions)**

Over the past 24hrs, how much has chronic pain affected your…

1. General activity

0 – 1 – 2 – 3 – 4 – 5 – 6 – 7 – 8 – 9 – 10

Does not interfere Completely Interferes

1. Mood

0 – 1 – 2 – 3 – 4 – 5 – 6 – 7 – 8 – 9 – 10

Does not interfere Completely Interferes

1. Walking ability

0 – 1 – 2 – 3 – 4 – 5 – 6 – 7 – 8 – 9 – 10

Does not interfere Completely Interferes

1. Normal work (includes work both outside the home and housework)

0 – 1 – 2 – 3 – 4 – 5 – 6 – 7 – 8 – 9 – 10

Does not interfere Completely Interferes

1. Relations with other people

0 – 1 – 2 – 3 – 4 – 5 – 6 – 7 – 8 – 9 – 10

Does not interfere Completely Interferes

1. Sleep

0 – 1 – 2 – 3 – 4 – 5 – 6 – 7 – 8 – 9 – 10

Does not interfere Completely Interferes

1. Enjoyment of life

0 – 1 – 2 – 3 – 4 – 5 – 6 – 7 – 8 – 9 – 10

Does not interfere Completely Interferes

**Empowerment (24 questions)**

1. I am capable of handling my condition
   1. Strongly Disagree
   2. Disagree
   3. Neither
   4. Agree
   5. Strongly Agree
2. I am satisfied with the level of health care information that I have available to me
   1. Strongly Disagree
   2. Disagree
   3. Neither
   4. Agree
   5. Strongly Agree
3. I can minimize the impact of my symptoms on my life
   1. Strongly Disagree
   2. Disagree
   3. Neither
   4. Agree
   5. Strongly Agree
4. I have the skills that help me feel in control of my condition
   1. Strongly Disagree
   2. Disagree
   3. Neither
   4. Agree
   5. Strongly Agree
5. Knowing more about my condition helps me manage it
   1. Strongly Disagree
   2. Disagree
   3. Neither
   4. Agree
   5. Strongly Agree
6. I can live a normal life despite my condition
   1. Strongly Disagree
   2. Disagree
   3. Neither
   4. Agree
   5. Strongly Agree
7. I participate in decisions concerning my health care
   1. Strongly Disagree
   2. Disagree
   3. Neither
   4. Agree
   5. Strongly Agree
8. I sometimes take health information to my doctor
   1. Strongly Disagree
   2. Disagree
   3. Neither
   4. Agree
   5. Strongly Agree
9. I understand my condition
   1. Strongly Disagree
   2. Disagree
   3. Neither
   4. Agree
   5. Strongly Agree

*How confident are you that you can ...*

*1 Being not at all confident to 10 being totally confident*

1. ... do all the things necessary to manage your condition on a regular basis?
   1. 0
   2. 1
   3. 2
   4. 3
   5. 4
   6. 5
   7. 6
   8. 7
   9. 8
   10. 9
   11. 10
2. ... judge when the changes in your illness mean you should visit a doctor
   1. 0
   2. 1
   3. 2
   4. 3
   5. 4
   6. 5
   7. 6
   8. 7
   9. 8
   10. 9
   11. 10
3. ... reduce the emotional distress caused by your health condition so that it does not affect your everyday life?
   1. 0
   2. 1
   3. 2
   4. 3
   5. 4
   6. 5
   7. 6
   8. 7
   9. 8
   10. 9
   11. 10
4. ... do things other than just taking medication to reduce how much your illness affects your everyday life?
   1. 0
   2. 1
   3. 2
   4. 3
   5. 4
   6. 5
   7. 6
   8. 7
   9. 8
   10. 9
   11. 10

*Over the past 6 months during visits with my palliative care doctor, I was… almost never / generally not / sometimes /most of the time / always*

1. asked for my ideas when we made a treatment plan
   1. almost never
   2. generally not
   3. sometimes
   4. most of the time
   5. always
2. asked to talk about any problems with my medicines or their effects
   1. almost never
   2. generally not
   3. sometimes
   4. most of the time
   5. always
3. satisfied that my care was well organized
   1. almost never
   2. generally not
   3. sometimes
   4. most of the time
   5. always
4. shown how what I did to take care of myself influenced my condition
   1. almost never
   2. generally not
   3. sometimes
   4. most of the time
   5. always
5. helped to make a treatment plan that I could carry out in my daily life
   1. almost never
   2. generally not
   3. sometimes
   4. most of the time
   5. always
6. helped to plan ahead so I could take care of my condition even in hard times
   1. almost never
   2. generally not
   3. sometimes
   4. most of the time
   5. always

*The last time you saw a doctor, how good was the palliative care doctor at each of the following?*

*Very good/ good/ neither/ poor/very poor/ does not apply*

1. asking about your symptoms
   1. Very good
   2. Good
   3. Neither
   4. Poor
   5. Very Poor
   6. Does not apply
2. listening to you
   1. Very good
   2. Good
   3. Neither
   4. Poor
   5. Very Poor
   6. Does not apply
3. explaining tests and treatments
   1. Very good
   2. Good
   3. Neither
   4. Poor
   5. Very Poor
   6. Does not apply
4. involving you in decisions about your care
   1. Very good
   2. Good
   3. Neither
   4. Poor
   5. Very Poor
   6. Does not apply
5. treating you with care and concern
   1. Very good
   2. Good
   3. Neither
   4. Poor
   5. Very Poor
   6. Does not apply
6. taking your problems seriously
   1. Very good
   2. Good
   3. Neither
   4. Poor
   5. Very Poor
   6. Does not apply

**COVID-19 (2 questions)**

1. How much is/did COVID-19 (coronavirus) impact your day-to-day life?
   - - Not at all
     - A little
     - Much
     - Very Much
     - Extremely
     - Decline to answer
2. Which of the following are you experiencing (or did you experience) during COVID-19 (coronavirus)? (check all that apply)

__ being diagnosed with COVID-19

__ fear of getting COVID-19

__ fear of giving COVID-19 to someone else

__ worrying about friends, family, partners, etc.

__ stigma or discrimination from other people (e.g., people treating you differently because of your identity, having symptoms, or other factors related to COVID-19)

__ personal financial loss (e.g., lost wages, job loss, investment/retirement loss, travel-related cancelations)

__ not having enough basic supplies (e.g., food, water, medications, a place to stay)

__ more anxiety

__ more depression

__ more sleep, less sleep, or other changes to your normal sleep pattern

__ increased alcohol or other substance use

__ feeling that I was contributing to the greater good by preventing myself or others from getting COVID-19

__ getting emotional or social support from family, friends, partners, a counselor, or someone else

__ getting financial support from family, friends, partners, an organization, or someone else
