# Supplementary material for: Co-created Mobile Apps for Palliative Care Using Community-Partnered Participatory Research: Development and Usability Study
Source: JMIR Form Res. 2022 Jun 23;6(6):e33849. doi: 10.2196/33849 (PMC9264134; doi:10.2196/33849)
Supplement: Multimedia Appendix 3 [file formative_v6i6e33849_app3.docx]

Patients consented/registered = 11

Baseline survey completion = 9

Age

Median: 58

Range: 49-82

Gender

Female (7)

Male (4)

Cancer Diagnosis

Breast (4)

Lung (3)

Colorectal (2)

Gastric (1)

Multiple Myeloma (1)

Race/Ethnicity

White/Caucasian (8)

Asian (1)

Education Level

Some college, no degree (1)

Four year college/university degree (5)

Postgraduate or professional degree (3)

Brief Pain Inventory – Interference Score

Daily Activities: 7.22 (4-10)

Mood: 6.89 (range 3-10)

Walking Ability: 6.00 (range 0-9)

Work: 6.56 (range 0-10)

Relations: 5.00 (range 0-9)

Sleep: 6.78 (range 2-10)

Enjoyment of Life: 7.33 (range 0-10)

Composite Average: 6.54 (High)

Empowerment Scale

Self management: 7.88 (range 4-10)

Judging needs for medical attention: 8.25 (range 5-10)

Ability to reduce illness-related emotional distress: 6.87 (range 4-10)

Ability to reduce illness-related symptoms w/o medication: 7.88 (range 5-10)

Digital Health Preferences/Values

*“Overall, how interested would you be to learn more about how to use mobile technologies to improve your health?*

Very Interested: 4

Moderately Interested: 5

*Which of the following statements most matches how you feel about your cell phone, even if neither one is exactly right?*

Not always need: 2

Couldn't live without: 7

---

Freedom: 6

Leash: 3

---

Connecting: 8

Distracting: 1

---

Helpful: 8

Annoying: 1

*How often do you use a computer for the following tasks?*

To receive or send email

Never: 1

Less than Once per Month: 0

Monthly: 0

Weekly: 1

Daily: 7

To order goods or services

Never: 1

Less than Once per Month: 0

Monthly: 2

Weekly: 5

Daily: 1

To read about general health information

Never: 2

Less than Once per Month: 0

Monthly: 0

Weekly: 4

Daily: 3

To read information about medications

Never: 2

Less than Once per Month: 0

Monthly: 4

Weekly: 2

Daily: 1

To read about my health conditions (for example, blood pressure, diabetes, or weight)

Never: 2

Less than Once per Month: 2

Monthly: 1

Weekly: 3

Daily: 1

To track my health conditions

Never: 2

Less than Once per Month: 1

Monthly: 0

Weekly: 5

Daily: 1

To track my diet or exercise

Never: 4

Less than Once per Month: 0

Monthly: 1

Weekly: 1

Daily: 3

To find out about services offered for my health conditions

Never: 2

Less than Once per Month: 4

Monthly: 0

Weekly: 1

Daily: 2
